# Hepatitis C Virus–Pediatric and Adult Perspectives in the Current Decade

**DOI:** 10.3390/pathogens14010011

**Published:** 2024-12-29

**Authors:** Nanda Kerkar, Kayla Hartjes

**Affiliations:** Massachusetts General Brigham for Children, 175 Cambridge Street, Boston, MA 02114, USA; khartjes@mgb.org

**Keywords:** HCV, children, immune response, epidemiology, vaccine, access to care, global elimination

## Abstract

Hepatitis C virus (HCV) infects both pediatric and adult populations and is an important cause of chronic liver disease worldwide. There are differences in the screening and management of HCV between pediatric and adult patients, which have been highlighted in this review. Direct-acting antiviral agents (DAA) have made the cure of HCV possible, and fortunately, these medications are approved down to three years of age. However, treatment in the pediatric population has its own set of challenges. The World Health Organization (WHO) has made a pledge to eliminate HCV as a public health threat by 2030. Despite this, HCV continues to remain a global health burden, leading to cirrhosis as well as hepatocellular carcinoma, and is a reason for liver transplantation in the adult population. Although rare, these complications can also affect the pediatric population. A variety of new technologies t have become available in the current era and can advance our understanding of HCV are discussed. Artificial intelligence, machine learning, liver organoids, and liver-on-chip are some examples of techniques that have the potential to contribute to our understanding of the disease and treatment process in HCV. Despite efforts over several decades, a successful vaccine against HCV has yet to be developed. This would be an important tool to help in worldwide efforts to eliminate the virus.

## 1. Introduction

Hepatitis C virus is an important cause of chronic liver disease in the pediatric and adult population [1]. It is associated with cirrhosis and hepatocellular carcinoma, leading to significant morbidity and mortality. In the pediatric population, the most common mode of transmission is perinatal [2]. HCV has been the most frequent indication for liver transplantation in adults in the USA. With the advent of direct-acting agents (DAA), cure of HCV is now possible. Despite this, HCV remains a burden in healthcare in the USA and globally. In this review, the differences between pediatric and adult HCV with respect to clinical presentation, screening, and management are highlighted. The epidemiology of HCV, distribution of different genotypes worldwide, and management of HCV in pediatric and adult populations are discussed. The goal of eliminating the virus by 2030, as proposed by the World Health Organization, is challenging, particularly in low-income countries [3]. The coronavirus epidemic has impacted the distribution of resources in recent years, and the focus needs to be brought back to eliminating HCV.

## 2. Epidemiology

**Figure 1:**
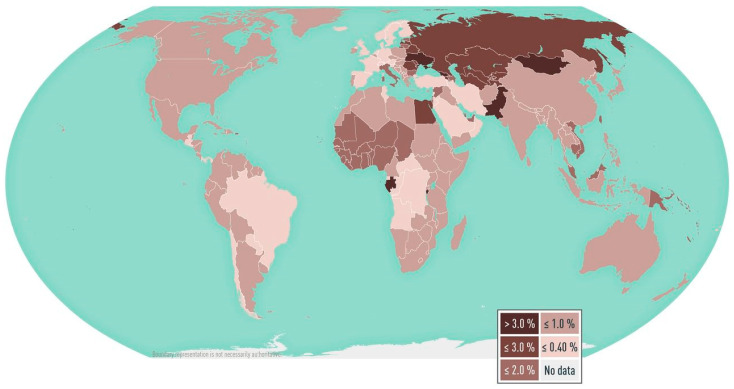
Worldwide_prevalence_of_hepatitis_C_viremia_2019. Disease data source: 2019 estimates of hepatitis C virus disease burden. CDA Foundation Polaris Observatory.

HCV was first identified as a cause of non-A non-B transfusion hepatitis in 1989 [4]. A homolog of HCV, hepacivirus, was initially identified in dogs in 2011, but was later shown to be widely prevalent in horses [5]. This is important as it may allow the development of suitable animal models to study HCV, given the inability to use chimpanzees for ethical reasons. There has been an epidemic of HCV in Western countries over the last 100 years [6]. Natural history and epidemiological studies have shown that global dissemination, particularly of subtypes 1a and 1b, could be an unintended consequence of medical treatments, blood transfusions, and injection drug use during World War II, leading to accelerated spread of the virus. The prevalence of pediatric HCV worldwide is 0.87% [7] vs. 2.5% worldwide for adults [8]. Currently, the mode of transmission in high-income countries is in the ‘at risk’ population, including intravenous drug users who share needles, men who have sex with men, and prisoners. In low-income countries, healthcare-associated transmission, including during invasive procedures, dental procedures, and blood transfusions, remains a frequent mode of transmission [9]. It is estimated that 40% of new HCV infections are associated with unsafe injections globally [10]. Although HCV reinfection is low after treatment, it is common in people entering Catalan prison [11]. The burden of HCV is disproportionately high in low- and middle-income countries (LMIC) [9]. Regions estimated to have a high prevalence of HCV in the general population (>3.5%) are Central and East Asia; and North Africa/Middle East; those with a moderate prevalence (1.5–3.5%) include South and Southeast Asia; Sub-Saharan Africa; Andean, Central, and Southern Latin America; the Caribbean; Oceania; Australasia; and Central, Eastern and Western Europe; whereas low prevalence [12]. Figure 1 depicts the global prevalence of HCV.

In the US, 3.5 million people are living with HCV, and more than 50% are unaware that they have HCV [13]. In 2022, the Center for Disease Control (CDC) report showed there were 67,400 estimated acute HCV infections [14]. There were 93,805 cases of newly reported chronic HCV and 12,717 HCV-related deaths were reported in 2022. The number of acute HCV was twice that seen in 2015, but the rate of acute HCV decreased by 6%. Perinatal transmission occurs in up to 10% of cases [15]. While the focus has been HCV in adults, at least 3.26 million children and adolescents (aged ≤ 18 years) are living with HCV infection worldwide.

As opposed to adults, where injection drug use is the most common mode of transmission, in pediatrics, the most common cause is vertical transmission. Transmission can occur both in utero and peripartum [16]. The risk of vertical transmission from mother to infant is 5.8% and increases to 10.8% in those who are coinfected with HIV [7]. Other factors that increase the risk of vertical transmission include HCV coinfection, maternal IV drug use, and likely higher HCV viral load; genotype and mode of delivery have not been found to increase the risk of transmission [8]. There have been other postulated reasons for the increase in HCV transmission, including prolonged rupture of membranes and invasive fetal monitoring, such as scalp electrodes, but the data are conflicting [17]. However, breastfeeding has not been found to increase transmission; thus, mothers who are infected with HCV are still encouraged to breastfeed, except to potentially be paused if nipples are cracked [18]. There is an increase in HCV among reproductive-age females, thus potentially further increasing the risk in the pediatric population [19]. This has led to the CDC’s recommendation to screen all pregnant women during each pregnancy for hepatitis C [16]. Similar to injection drug use being the most common mode of HCV transmission in adults, injection drug use in adolescents is a significant cause of HCV, with concerns about increasing drug use and, hence, HCV in this population [16,17,18].

Worldwide, HCV is highly diverse and has been divided into seven genotypes and several subtypes. Genotypes 1 and 2 are predominant in the West—Americas, Europe, and Japan. Genotypes 3 and 6 are most common in South and Southeast Asia, while genotypes 4, 5, and 7 are seen in Africa and are now spreading to Europe [20]. Egypt is the nation most affected by HCV. The predominant genotype is 4, which is observed in 94% of patients with HCV [21]. Despite the large numbers, the prevalence in Egypt appears to be declining, which is consistent with a contracting epidemic. Estimated HCV infection rates in the general population in the Asia-Pacific region were 0.1–14.7% [9]. In a retrospective multicenter study that enrolled 11,008 samples collected between August 2018 and July 2019 from 29 provinces/municipalities in China, HCV subtypes 1b and 2a remained the most common subtypes in the Chinese mainland, and their proportions decreased over the past years, while the proportions of genotypes 3 and 6 increased [22].

## 3. Natural History and Clinical Features

Children have a higher chance of spontaneously clearing HCV infection, especially if it is acquired vertically. If acquired perinatally, children have a 20–40% chance of clearing by the age of five [19]. In adults, 20% may clear HCV infection within 12 weeks of infection [23]. Typically, disease progression is slow in children, and very few pediatric patients require liver transplants [2]. However, although less common than in the adult population, there are cases of children progressing to cirrhosis and hepatocellular carcinoma. A large retrospective study conducted in the UK found that 32% of patients developed liver disease; however, the median age was 33 years after infection and 5% of patients developed hepatocellular carcinoma [24]. However, a large multicenter observational study from Japan demonstrated no episodes of hepatocellular carcinoma or cirrhosis in any of the children during the follow-up period [25]. Cases of pediatric patients with HCV infection who develop cirrhosis have been reported. In a multicenter study out of Italy, six of 332 cases progressed to decompensated cirrhosis at a mean age of 9.6 years [26]. In addition, the Peds-C Trial, which was designed to examine the treatment of children with HCV with peginterferon and ribavirin, examined 121 liver biopsies prior to treatment. Two of these patients had cirrhosis on liver biopsy, and none of these patients had signs of hepatic decompensation [27]. In 2000, a center in New York reported children with decompensated cirrhosis from perinatally acquired HCV at 4, 6, and 11 years of age [28]. Thus, although not typically as common as in adults, HCV can progress in young children, and lifetime infection with HCV can certainly lead to fibrosis and hepatocellular carcinoma in adults. A systematic review found that 16% of adults 20 years after HCV develop cirrhosis [29]; however, studies have deferring rates on the exact percentages of progression to cirrhosis. HCV is one of the main drivers of the development of hepatocellular carcinoma in adults; however, it typically only occurs in those with cirrhosis due to HCV infection. Thus, even after attaining sustained viral clearance, the risk of hepatocellular carcinoma development continues in patients with cirrhosis [30]. The percentage of adults undergoing liver transplantations due to HCV has fallen greatly over the years to as low as 4.4% in 2022, and in children, HCV is a rare indication for liver transplantation [31].

Although HCV infects both pediatric and adult patients, the approach to diagnosis and clinical care of pediatric patients differs from that of adult patients. Table 1 outlines the main differences and similarities between HCV infections in the pediatric vs. the adult population.

## 4. Management

Prior to age three, treatment is deferred, given that children may clear the infection on their own [38]. Although treatment is approved from age three, it is often deferred until a patient can reliably take the medication, given the risk of resistance development with improper use of the medication. Pangenotypic treatments approved in children include sofosbuvir–velpatasvir for twelve weeks or glecaprevir–pibrentasvir for eight weeks, assuming the patient is treatment-naïve without decompensated cirrhosis. Ledipasvir/sofosbuvir for twelve weeks is also approved in the pediatric population for genotypes 1, 4, 5, and 6 [38]. Clinical trials have demonstrated the safety of these drugs in pediatric populations. Most patients in clinical trials were treatment-naïve, although a small percentage were treatment-experienced. In addition, most studies did not include cirrhotic patients except for ledipasvir–sofosbuvir, which enrolled one patient with cirrhosis [32,34,39]. The cure rate is high when adherence to medication is obtained, hence why in pediatric patients, given the lower rates of cirrhosis development, treatment is often deferred until patients can reliably take medications. However, for those who do not swallow pills, granule formations are available.

Like pediatric patients, in adult patients sofosbuvir–velpatasvir for twelve weeks or glecaprevir–pibrentasvir for eight weeks is preferred in patients without decompensated cirrhosis [37]. If compensated cirrhosis is present, glecaprevir–pibrentasvir for eight weeks is the preferred treatment, and sofosbuvir/velpatasvir is used, except it is avoided in genotype 3 unless resistance testing is completed [33]. However, in decompensated cirrhosis, patients should be referred to be cared for in a liver center as not all these patients benefit from DAA therapy. However, if they are ribavirin-eligible, all genotypes can be treated with sofosbuvir/velpatasvir with ribavirin; for genotypes 1, 4, 5, and 6, ledipasvir/sofosbuvir with ribavirin can be used. If patients are ribavirin ineligible, treatment can be performed as above; however, without ribavirin and for twenty-four weeks as opposed to twelve weeks [40].

The Infectious Disease Society of America (IDSA), along with the American Association of the Study of Liver Disease (AASLD) have published guidelines on the treatment of specialized patients with HCV, including pregnant women and those with concurrent HIV. All pregnant women should be tested for HCV infection during each pregnancy [41]. Although it is best to treat HCV prior to pregnancy, when HCV is discovered during pregnancy, two small clinical trials found that ledipasvir/sofosbuvir was safe during pregnancy [42,43]. However, given the lack of data, there is no recommendation, and treatment can be considered on a case-by-case basis. Those with HIV/HCV coinfection are treated the same as those without HIV infection; however, interactions with antiretroviral medications have to be screened for [40].

Given the difference in the mode of transmission between children and adults, screening guidelines are different. The CDC recommends screening all children born to an HCV-positive mother, those whose mothers have an unknown HCV RNA status with a positive HCV antibody, or a mother with a history of IV drug use between the ages of two and six months of age with nucleic acid testing [19]. Although children of this age are not treated, this recommendation was made to give more than half of the perinatally infected children not being linked into care. If testing is positive, children should be referred to an HCV specialist, and siblings with the same biological mother should also be screened [2]. Other children that should be tested include international adoptees or refugees, IV drug users, children with chronic elevation in AST/ALT, those with HIV infection, and children who were sexually assaulted [2]. The CDC now recommends that all adults 18–79 years of age be screened at least once for HCV [44]. In those with continued risk for HCV infection, such as IV drug use or men who have sex with men who also have HIV, recommendations are to screen approximately yearly [40]. The CDC also recommends that pregnant women be screened during every pregnancy [44].

The screening guidelines for both adults and children are vital, given that both children and adults with HCV are typically asymptomatic. HCV is usually detected during screening, as opposed to during laboratory work performed for symptoms. However, children rarely present with abdominal pain, hepatomegaly, or fatigue [45], whereas adults can present with symptoms such as scleral icterus, jaundice, and right upper quadrant pain [46]. Once found to be positive for HCV, it is vital that children are not excluded from day care or other household activities. Adults should not be excluded from work. HCV does not spread if appropriate blood-borne precautions are taken.

## 5. HCV and Vaccine Development

There have been many advancements in the field of HCV with the development of rapid molecular techniques, improvements in blood screening, and most importantly, the development of oral direct-acting antiviral (DAA) agents that can cure HCV. In 2021, the Nobel Prize was awarded to three scientists, Michael Houghton, Charles Rice, and Harvey Alter, for their role in the discovery and characterization of HCV [47]. Despite tremendous research and advances, it has still not been possible to develop an effective preventive vaccine against HCV.

The hepatitis C virus is a spherical enveloped virus approximately 50 nm in diameter and is a member of the Flaviviridae (Hepacivirus) family [48]. Its genome is a positive single-stranded RNA [4] that is unsegmented and approximately 9.5 kb in size. The most important characteristic of the HCV genome is its sequence heterogeneity (quasispecies), although it is not uniform across the genome. The primary driver of HCV diversity is RNA-dependent RNA polymerase, which is prone to error [49]. The heterogeneous portions of the genome are the genes encoding envelope proteins, and the most heterogeneous region, located at the 5′ end of the E2 gene, is named the first hypervariable region (HVR1). Neutralizing antibodies are thought to be directed against this site [50,51].

HLA class I restricted cytotoxic T lymphocytes (CTL) provide a major defense mechanism against viral infections. The common chimpanzee *Pan troglodytes* was previously used as an animal model for human HCV [52]. Experiments during acute HCV (<20 weeks) infection showed that infection was cleared in those with a strong CTL response versus staying weak in those who developed chronic HCV [53]. These studies were reproduced in human subjects using HLA-tetramers and an IFN-gamma enzyme-linked immunospot [54]. It has also been shown that the strength of the T-cell response in the early stages of infection may be a critical determinant of disease resolution and control of infection [55]. Weaker CTL responses may allow virus variants to emerge that escape immune recognition. Studies on chronic HBV and HIV infections [56,57] have demonstrated that escape is a possible event when the CTL response is focused on a single or few immunodominant epitopes. In five HLA-A2-positive patients with acute HCV, it was noted that virus variants did not appear in three patients who recovered, but in the remaining two patients who developed chronic HCV, altered peptide ligands capable of antagonizing CTL activity emerged rapidly [58]. In immune chimpanzees, the removal of CD8 T cells resulted in a prolonged period of viral replication [59], while the depletion of CD4 T cells resulted in low levels of persistent viremia lasting several months [60].

There is also evidence of the role of humoral immunity in the control of HCV. Clearance of the virus spontaneously after acute HCV has been associated with the presence of neutralizing antibodies that recognize multiple heterologous strains [61,62,63]. Polyclonal immunoglobulin from a chronically infected donor prevented infection in human liver chimeric mice [64,65]. Despite this and other data showing the role of humoral and CTL responses in the spontaneous clearance of HCV, the virus itself, by continuously replicating and maintaining sequence heterogeneity, continues to evade the host defense system. The immune responses in individuals who had spontaneously cleared the virus were the basis for developing a vaccine that progressed to phase 2 clinical trials in humans. Although the regimen stimulated HCV-specific T cell responses in the majority of vaccinees in a cohort of IVDU, no reduction in the incidence of chronic infection was observed [66]. Going forward, continued research and funding for the development of an effective vaccine against HCV are essential to move toward the goal of global eradication.

## 6. Global Elimination of Hepatitis C

In 2013, the (AASLD and the IDSA collaboratively developed evidence-based guidance for the diagnosis and management of HCV, and the information has been regularly updated [40]. The information is freely accessible through a web-based platform (www.hcvguidelines.org), and since its launch, it has been accessed by millions of users from 201 countries. In 2016, the efficacy of the DAA led the World Health Organization (WHO) to make a pledge to eliminate HCV as a public health threat by 2030. Organizations around the world, including the European Association of the Study of Liver and the Asian Pacific Association of the Study of Liver, have developed strategies to combat HCV. Unfortunately, the global pandemic with severe acute respiratory syndrome coronavirus 2 infection has caused major interruptions to the HCV elimination plan. Resources worldwide have focused on managing the new epidemic, which has become the primary focus. In the US, telemedicine was incorporated into clinical workflows, and much preventive care, including HCV testing, was not routinely performed to preserve resources to combat coronavirus disease 2019 (COVID-19) [67]. A study investigating HCV screening at Boson Medical Center revealed that hospital-wide total HCV testing decreased by 49.6% and new HCV+ patient identification decreased by 42.1%. In ambulatory clinics, testing decreased by 71.9%, and new HCV+ identification decreased by 63.3% [67].

Even though DAA are available, only one in three is cured of HCV in the USA. With the pandemic behind us, in March 2023, an initiative was made in the US to work on HCV. The fact that HCV is highly prevalent in those who are incarcerated, those who inject drugs, and low-income families is a barrier to diagnosis and treatment globally [42]. Stigma in health care has been shown to be related to breaches in confidentiality, inappropriate assumptions about those who become infected, and undue fears of becoming infected [68]. Participants in the study acknowledged shame, fear of stigmatization, and extreme caution around disclosing their diagnosis of HCV. Destigmatizing the disease through education and public health campaigns is necessary to allow healthcare professionals to work effectively and gain the trust of the community with which they must work. In pediatric HCV, which is usually the result of perinatal transmission, there is increased caregiver stress in the family [69]. There is no legal requirement to disclose HCV in the United States. The CDC and patient advocacy groups suggest that this be disclosed to sexual partners when appropriate. Hepatitis testing for diagnosis must always be voluntary, consent for testing must be informed by pre-test information, and it must be linked to prevention, treatment, care, and support services to maximize both individual and public health benefits [3].

There are many reasons for HCV being a bigger problem in LMIC, including the fact that there is a lack of awareness both in the general population and in health care workers about HCV. There is also stigma and discrimination in the high-risk population. The lack of infrastructure for testing for HCV and limited access to viral load tests in these countries is a rate-limiting factor. There is a lack of surveillance programs, and most LMICs do not have national viral hepatitis strategies. Action to combat viral hepatitis has now been integrated into the United Nations 2030 Agenda for Sustainable Development. From a public health perspective, HCV diagnosis and care could be simplified to just two visits: (i) diagnosis of active HCV infection and standardized treatment regardless of disease stage, and (ii) confirmation of cure post-treatment completion [70]. In LMIC, where HCV accounts for 80% of the global burden, it is important for people to have access to a simple, affordable diagnostic test for HCV that is available at the point of care. This will allow the identification of HCV and then therapy all in one visit, and it may be the only way to eliminate the virus in LMIC and globally. The cost of treatment remains a barrier. In the US, it was noted that the cure rate was aligned with the ability to pay, raising the issue that this matter could be resolved with a central payer system [71]. For LMIC, in a memorandum of understanding, it was proposed to reduce the cost of antiviral drugs in HCV by 90% to align with the reduction in cost for treatment of hepatitis B and HIV, as has been achieved with Tenofovir [72].

### Future Directions

Artificial intelligence algorithms can substantially increase the effectiveness of HCV screening [73]. Machine learning algorithms could also identify risk factors that led to treatment failure with DAA and have the potential to inform public health policies regarding the curative treatment of HCV [74]. Liver organoids are defined as a 3D structure derived from pluripotent stem cells, progenitor, and/or differentiated cells, as well as primary and metastatic tumors, which self-organize through cell-cell and cell-matrix interactions to recapitulate the architecture and functional features of the tissue of origin (a). The organoids can model human liver disease, including viral hepatitis, and offer an exciting opportunity to better understand the disease process [75,76]. Another emerging technology is liver-on-chip. This is a novel liver cell culture platform that mimics the key structure and core functions of the liver [77]. The liver-on-chips consist of real living human hepatocytes, and those commonly used include primary human cells and immortalized human cell lines. Hepatocytes are seeded in engineered systems that reliably recapitulate liver structure and function [77]. There are many types of liver-on-chip, and although there are shortcomings, they have the potential to overcome hurdles in animal models and other systems.

## 7. Summary

In summary, HCV is an important cause of chronic liver disease in pediatric and adult populations and is associated with significant morbidity and mortality. It is a cause of cirrhosis and hepatocellular carcinoma and is an indication for liver transplantation in the adult population. The differences in screening and management between adult and pediatric HCV have been highlighted. The development of DAA has changed the landscape in HCV. HCV is now curable in both pediatric and adult populations. However, despite this, there continues to be an HCV burden worldwide, which is mainly driven by poor access to resources and a lack of awareness of the problem. Given the high cure rate of HCV, if access to care can be improved and the stigmata of HCV removed by education and counseling, there is hope for worldwide elimination of HCV in both pediatric and adult populations. The need for internationally coordinated surveillance for HCV and reduced pricing of medications to treat HCV, particularly in LMIC, has become necessary if the goal of HCV elimination by 2030 is to be realized. Artificial intelligence is an important tool in the armamentarium against HCV and can be utilized in myriad ways to speed up progress in primary prevention, surveillance, and effective therapy.

## Figures and Tables

**Table 1:** Comparisons between Pediatric and Adult HCV.

	Pediatrics	Adults
Prevalence of disease worldwide	0.87% [4]	2.5% [5]
Common mode of transmission	- Most common mode of transmission is mother to infant during the perinatal period [2,11]. IV drug use in older children [11,12,13] Less common modes of transmission include: - Contaminated tattoos - Intranasal cocaine use - High-risk sexual behavior - Household transmission	- Most common mode of transmission is Injection drug use [9] Less common modes of transmission include: - Blood transfusion or organ transplantation - Sexual transmission is controversial - Household transmission [9]
Clinical Presentation	Patients are typically asymptomatic. However, they can have fatigue, abdominal pain, hepatomegaly [32]	Patients are typically asymptomatic. However, symptoms such as jaundice and right upper quadrant pain can develop 2–26 weeks after exposure [33]
Screening	- Children born to a: HCV-positive mother, a mother with a history of IV drug use, or a mother with a positive HCV antibody with unknown HCV RNA should be tested with HCV RNA between two and six months of age [14] - International adoptees or refugees should be screened for HCV starting with HCV antibodies, as well as IV drug users, children with chronic elevation in AST/ALT, those with HIV infection, and children who were sexually assaulted [19].	- Patients 18 years old or greater should be screened at least once with HCV antibodies [34] - If there is concern for acute HCV infection, both HCV antibody and HCV RNA should be sent. - Pregnant individuals [11] - If there is continued risk for HCV exposure, the HCV antibody should be sent approximately every 12 months [35]. - If the patient is immunocompromised, consider sending HCV RNA with HCV antibody, given the concern for a potential false negative antibody result [36]
Percentage of spontaneous clearance	- 20–40% if acquired perinatally, typically by age 5 [14].	- Approximately 20% - If spontaneously clear, it is typically within 12 weeks of infection [35].
Treatment of Acute HCV	There is no data in pediatrics; most patients are treated similarly to chronic hepatitis.	Patients are treated the same as chronic hepatitis [35].
Treatment Chronic HCV	- Treatment is deferred until at least age three [28] - After age three, treatment is deferred until the patient can reliably take the medication - Treatment in those without decompensated cirrhosis: Sofosbuvir-velpatasvir for 12 weeks [36,37] Glecaprevir-pibrentasvir for 8 weeks [28,37]	- Without decompensated cirrhosis besides genotype 3: - Sofosbuvir-velpatasvir for 12 weeks [36] - Glecaprevir-pibrentasvir for 8 weeks [36] - With compensated cirrhosis and genotype 3 - glecaprevir–pibrentasvir for eight weeks [36] - Decompensated cirrhosis - Undergo liver transplantation evaluation if MELD > 10. Timing of treatment depends on whether the transplant candidate [35]
Counseling	Patients should be counseled to avoid sharing razors, toothbrushes, and nail clippers. They should also avoid high-risk behaviors such as self-piercing, tattooing, and IV drug use. Children should not be excluded from daycare or household activities.	There is no difference in the counseling between Pediatric and adult patients.
Development of liver disease and cirrhosis	There is typically a slow progression to fibrosis. Advance disease is uncommon before adulthood; however, it can be seen in children. Obesity, HIV, and HBV are some of the risk factors for severe disease [19,22,23,24].	The risk of cirrhosis increases with the length of time of infection. HCV accounts for 1/3 of HCC cases in the US. However, HCC almost only occurs in those with cirrhosis [25,26].
Liver transplantation for HCV-related liver disease	Rare indication	4.4% of adult liver transplants are those w/HCV [27]

## Abbreviations

HCV, hepatitis C virus; CDC, Center of Disease Control; DAA, direct-acting agents; WHO, World Health Organization; CTL, cytotoxic T lymphocytes; IVDU, intravenous drug use; AASLD, American Association of the Study of Liver Disease; IDSA, Infectious Disease Society of America; LMIC, low- and middle-income countries.
